# Recent Progress in Self-Healing Triboelectric Nanogenerators for Artificial Skins

**DOI:** 10.3390/bios15010037

**Published:** 2025-01-10

**Authors:** Guoliang Li, Zongxia Li, Haojie Hu, Baojin Chen, Yuan Wang, Yanchao Mao, Haidong Li, Baosen Zhang

**Affiliations:** 1 Henan Energy Conversion and Storage Materials Engineering Center, College of Science, Henan University of Engineering, Zhengzhou 451191, China; 2 Key Laboratory of Materials Physics of Ministry of Education, School of Physics, Zhengzhou University, Zhengzhou 450001, China

**Keywords:** triboelectric nanogenerator, self-healing, artificial skin, tactile sensing, human–machine interface

## Abstract

Self-healing triboelectric nanogenerators (TENGs), which incorporate self-healing materials capable of recovering their structural and functional properties after damage, are transforming the field of artificial skin by effectively addressing challenges associated with mechanical damage and functional degradation. This review explores the latest advancements in self-healing TENGs, emphasizing material innovations, structural designs, and practical applications. Key materials include dynamic covalent polymers, supramolecular elastomers, and ion-conductive hydrogels, which provide rapid damage recovery, superior mechanical strength, and stable electrical performance. Innovative structural configurations, such as layered and encapsulated designs, optimize triboelectric efficiency and enhance environmental adaptability. Applications span healthcare, human–machine interfaces, and wearable electronics, demonstrating the immense potential for tactile sensing and energy harvesting. Despite significant progress, challenges remain in scalability, long-term durability, and multifunctional integration. Future research should focus on advanced material development, scalable fabrication, and intelligent system integration to unlock the full potential of self-healing TENGs. This review provides a comprehensive overview of current achievements and future directions, underscoring the pivotal role of self-healing TENGs in artificial skin technology.

## 1. Introduction

Artificial skin, inspired by the complex properties of human skin, is a transformative technology with applications in robotics, prosthetics, and healthcare [1,2,3,4,5,6,7,8]. By replicating the flexibility, sensitivity, and adaptability of biological skin, it enables advanced functionalities such as energy harvesting and tactile sensing [9,10,11,12,13,14,15,16]. Triboelectric nanogenerators (TENGs) have emerged as a promising technology in this domain due to their ability to convert mechanical energy into electrical signals. TENGs operate through four fundamental working modes: vertical contact-separation mode, lateral sliding mode, single-electrode mode, and freestanding triboelectric-layer mode, which enable energy harvesting based on different types of mechanical interactions [17,18,19,20,21,22,23,24,25,26]. However, the lack of damage tolerance in traditional TENGs poses a significant challenge, leading to performance degradation and limited operational lifespan [27,28,29,30,31]. The integration of self-healing materials into TENG addresses these issues by enabling autonomous repair, enhancing durability, and ensuring sustained functionality under repeated stress [32,33,34,35,36,37].

Self-healing TENGs are TENGs designed using self-healing materials that possess the intrinsic capability to recover their structural and functional properties after damage through dynamic molecular interactions. These materials enable the devices to maintain their performance over repeated damage and repair cycles, offering significant advantages for long-term applications. Self-healing TENGs employ materials such as polymers [38,39,40,41], nanocomposites [42,43,44], and hydrogels [45,46,47,48]. These materials restore functionality through dynamic bonding mechanisms, allowing devices to recover rapidly from mechanical damage while preserving their triboelectric performance. Structural innovations, including layered configurations and encapsulated designs, further enhance energy harvesting efficiency and mechanical stability. Additionally, the incorporation of nanomaterials like graphene and carbon nanotubes improves electrical conductivity and mechanical robustness, expanding the operational scope of TENGs in diverse environments [49,50,51,52,53]. These advancements facilitate multifunctional properties, such as stretchability and environmental resilience, which are critical for artificial skin applications.

**Figure 1:**
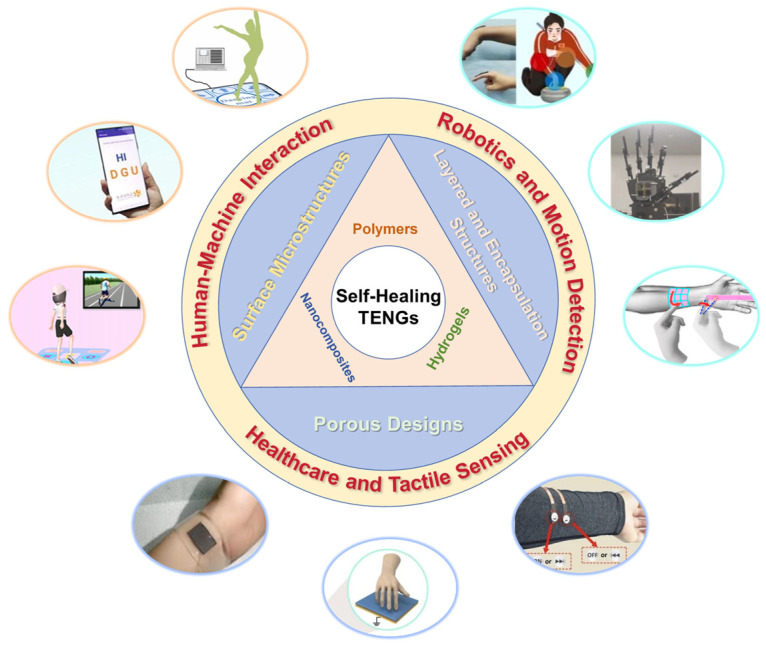
Recent advancements in self-healing TENGs for artificial skin applications. The focus is on innovations in self-healing materials, structural designs, and configurations that enhance performance, durability, and integration with flexible substrates. Practical applications of self-healing TENGs are presented, including their use in robotics, motion detection, human–machine interaction, tactile sensing, and healthcare. Reprinted with permission from Ref. [54]. Copyright 2024, Elsevier. Reprinted with permission from Ref. [55]. Copyright 2021, Elsevier. Reprinted with permission from Ref. [56]. Copyright 2021, Elsevier. Reprinted with permission from Ref. [57] Copyright 2023, American Chemical Society. Reprinted with permission from Ref. [58] Copyright 2022, Elsevier. Reprinted with permission from Ref. [59]. Copyright 2022, Elsevier. Reprinted with permission from Ref. [60] Copyright 2018, American Chemical Society. Reprinted with permission from Ref. [61] Copyright 2024, Elsevier. Reprinted with permission from Ref. [62]. Copyright 2024, Elsevier.

This review provides a detailed overview of the recent advancements in self-healing TENGs specifically designed for artificial skin applications. It explores the latest material innovations that have enhanced the performance, durability, and self-healing capabilities of TENGs, allowing these devices to autonomously repair themselves after damage, thereby extending their lifespan and reliability. This review also examines novel structural designs of self-healing TENGs, discussing how different configurations optimize the energy harvesting process, improve mechanical flexibility, and enable better integration with flexible substrates. In addition, the article highlights the practical applications of these self-healing TENGs, focusing on their potential use in a wide range of fields, including robotics, wearable electronics, prosthetics, and healthcare (Figure 1). Challenges such as scalability, durability, and environmental adaptability are critically analyzed, and future research directions are proposed. By addressing these aspects, this article underscores the transformative potential of self-healing TENGs, offering a blueprint for the development of next-generation artificial skin technologies.

## 2. Material Innovation in Self-Healing TENGs

### 2.1. Polymers

The application of self-healing polymers in TENGs has garnered significant attention in recent years. These materials can autonomously repair damage through specific mechanisms, thereby extending device longevity and maintaining high efficiency. Self-healing polymers used in TENGs can generally be categorized into two main types: elastomers and conductive polymers. Elastomers, such as silicones and polyurethanes, are widely used due to their remarkable self-healing capabilities. Silicones, known for their excellent thermal stability and electrical insulation properties, demonstrate the potential to enhance device longevity. Polyurethanes, on the other hand, are favored for their superior mechanical properties and outstanding recovery ability, making them ideal for devices requiring a balance between flexibility and mechanical strength. This balance is achieved through crosslinking mechanisms that enable structural repair and durability. Conductive polymers represent another critical category of self-healing polymers in TENG applications. The incorporation of conductive polymers not only improves the electrical conductivity of the material but also enhances its self-healing properties. Common conductive polymers include polyaniline (PANI) and polypyrrole (PPy). Through doping or composite strategies, they enhance TENG output performance by improving conductivity and self-healing efficiency. PANI offers tunable conductivity that can be optimized by adjusting doping levels. Additionally, its internal conductive network can self-repair after damage, ensuring functional integrity. PPy, renowned for its excellent chemical stability and mechanical properties, is extensively utilized in self-healing TENGs. It achieves functional recovery after damage through mechanisms such as self-assembly and hydrogen bonding.

**Figure 2:**
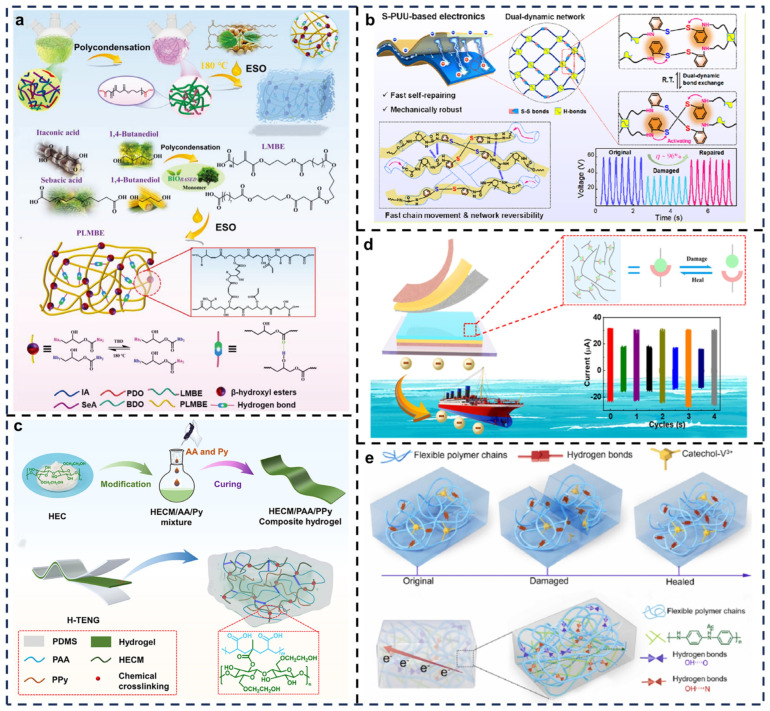
Preparation of self-healing polymers for TENGs. (**a**) Synthesis pathway of PLMBE. Reprinted with permission from Ref. [63]. Copyright 2023, Wiley. (**b**) Room-temperature self-healing nanogenerator enabled by a fast-reversible dual-dynamic network. Reprinted with permission from Ref. [64]. Copyright 2023, American Chemical Society. (**c**) Schematic diagram and preparation process of the hydrogel. Reprinted with permission from Ref. [65]. Copyright 2023, Elsevier. (**d**) TENGs based on synthesized linear organosilicon-modified polyurethane. Reprinted with permission from Ref. [66]. Copyright 2022, American Chemical Society. (**e**) Design of PMBEug-OH-V and PMBEug-OH-PANI polymers. Reprinted with permission from Ref. [67]. Copyright 2023, Elsevier.

To improve self-healing performance, Wang et al. designed a bio-based elastomer with hydrogen bonds and β-hydroxy esters (Figure 2a) [63]. The dynamic supramolecular polymer network formed by these hydrogen bonds effectively dissipates strain energy, acting as sacrificial bonds to enhance the mechanical performance of the elastomer. This material maintains high flexibility and excellent mechanical strength over a temperature range from −10 °C to room temperature. Unlike conventional elastomers, which tend to become brittle and lose elasticity at extremely low temperatures, the bio-based self-healing elastomer retains its energy conversion efficiency even under severe cold conditions. The TENGs fabricated with this material exhibit high output performance, particularly at −10 °C, making them suitable for energy harvesting applications in extreme climates.

To overcome the challenge of slow healing times, Cheng et al. developed a fast-reversible dual-dynamic network elastomer (Figure 2b) [64]. This innovative material balances self-healing capability and mechanical performance and has been successfully applied to elastomeric TENGs. The synthesized elastomer features a dual-dynamic network structure formed by disulfide-containing polyurethane-urea (S-PUU), with adjustable crosslinking density and high recoverability, enabling rapid self-healing at room temperature. By incorporating aromatic disulfide linkers into the hard segments of polyurethane, the material forms a dual-dynamic network comprising abundant reversible disulfide (S-S) and hydrogen bonds. This structure supports efficient healing across diverse environments, ensuring durability after repeated damage cycles.

For applications in wearable electronics and flexible energy harvesting devices, Sun et al. developed a self-reinforced conductive cellulose composite hydrogel (Figure 2c) [65]. The hydrogel combines hydroxyethyl cellulose macromonomer (HECM) with conductive polymers such as PPy. The hydrogel matrix serves as a flexible network, balancing conductivity and mechanical properties through a one-step self-assembly process. HECMs provide mechanical support, imparting the hydrogel with high elastic modulus and improving its tensile and compressive strength. Meanwhile, the incorporation of conductive polymers like PPy endows the hydrogel with excellent conductivity, which is crucial for signal transmission in sensor applications. This synergy between flexibility, conductivity, and mechanical robustness makes it particularly promising for next-generation flexible electronics.

To address performance degradation caused by corrosion in harsh environments, Sun et al. designed a self-healing TENG based on a polyurethane coating (Figure 2d) [66]. The TENG-generated electric energy provides cathodic protection potential to metal surfaces, reducing the oxidation rate and significantly enhancing corrosion resistance. A self-healing polyurethane elastomer containing dynamic disulfide and hydrogen bonds is synthesized, ensuring robust mechanical self-repairability. The polyurethane is further compounded with high-performance triboelectric fillers (e.g., polytetrafluoroethylene) and conductive enhancers (e.g., carbon nanotubes) to create a thin coating film through casting or spraying. The TENG is successfully applied to a self-powered cathodic protection system, harvesting ambient mechanical energy for long-term corrosion protection of metallic structures, such as steel. Its self-healing capabilities also extend the device’s lifespan by enabling damage recovery.

To overcome limitations in damage tolerance and healing capacity, Hou et al. developed a multifunctional triboelectric material combining durability and self-healing (Figure 2e) [67]. The flexible TENG consists of a triboelectric layer of PMBEug-OH-V-2-4 and an electrode layer of PMBEug-OH-PANI-20%, with polydimethylsiloxane (PDMS) elastomer serving as the negative triboelectric layer. When PDMS interacts with PMBEug-OH-V-2-4, differences in electron affinity generate equal but opposite charges at their interface. These charges induce dipole migration in PMBEug-OH-PANI-20%, maintaining electrostatic neutrality. The interface achieves a balanced state, allowing the TENG to convert mechanical energy into electricity through repeated contact and separation. This innovative material significantly improves TENG durability and energy conversion efficiency, broadening its potential applications in wearable electronics.

### 2.2. Hydrogels

Hydrogel materials are widely used in the field of self-healing electronic skin, with ongoing research and applications progressing rapidly toward high performance, multifunctionality, and environmental friendliness. Ion-conductive hydrogels, known for their remarkable flexibility, high water content, and ionic conductivity, have found extensive applications in self-healing TENGs. Their ability to adapt to complex deformation environments and support ionic conduction makes them suitable for efficient electrical energy output in TENGs. Representative examples, such as polyvinyl alcohol (PVA) and gelatin-based hydrogels, exhibit excellent stretchability and self-healing capabilities, enabling rapid recovery of mechanical and electrical properties even after damage. These properties make them ideal candidates for flexible electronic and wearable technologies.

Composite hydrogels further enhance performance by incorporating inorganic nanomaterials (e.g., graphene, silver nanowires) into hydrogel matrices. Such combinations retain the inherent flexibility and water content of hydrogels while improving tensile strength, toughness, and electrical properties. For instance, graphene imparts superior electronic conductivity to composite hydrogels, while silver nanowires enhance electrical stability and fatigue resistance, making them ideal candidates for high-performance flexible electrodes and sensing layers. The synergistic integration of these components positions hybrid hydrogels as promising materials for electronic skin and advanced wearable applications.

**Figure 3:**
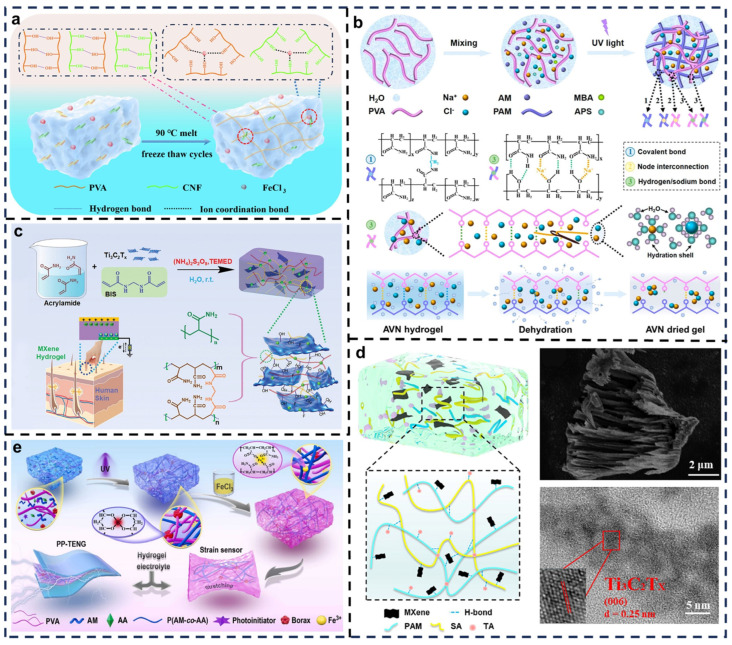
Synthesis of self-healing hydrogel materials in TENGs. (**a**) CNF-FeCl₃ hydrogel synthesized from PVA. Reprinted with permission from Ref. [68]. Copyright 2024, Elsevier. (**b**) Schematic illustration of AVN hydrogel. Reprinted with permission from Ref. [69]. Copyright 2021, American Chemical Society. (**c**) Preparation process and crosslinking mechanism of MPP–hydrogel. Reprinted with permission from Ref. [70]. Copyright 2022, Wiley. (**d**) SEM and TEM images of MXene. Reprinted with permission from Ref. [28]. Copyright 2023, American Chemical Society. (**e**) Design strategy schematic of PVA/P(AM-co-AA)-Fe^3+^ (DN) hydrogel. Reprinted with permission from Ref. [71]. Copyright 2022, Elsevier.

To meet the increasing demand for flexible electronic devices, researchers are focusing on developing high-performance, multifunctional hydrogels. Li et al. developed a cellulose nanofiber (CNF)-reinforced PVA-based ionic conductive hydrogel with high strength and fatigue resistance using a single-pot freeze–thaw method (Figure 3a) [68]. Traditional hydrogels are prone to fatigue under repeated stress, whereas CNF-reinforced PVA hydrogels exhibit excellent anti-fatigue properties, maintaining mechanical strength and stable electrical performance even after tens of thousands of loading cycles. This resilience addresses a common limitation of traditional hydrogels. The incorporation of CNFs, through physical and chemical crosslinking with the PVA network, effectively suppresses the propagation of microcracks within the hydrogel matrix, enhancing its tear resistance and fracture toughness. This improvement prolongs the hydrogel’s operational lifespan, making it suitable for long-term applications in flexible electronics.

One of the main challenges for hydrogel-based S-TENGs (HS-TENGs) is their susceptibility to dehydration, which narrows their operating temperature range and shortens their lifespan. Wu et al. proposed a sustainable ionic-conductive organohydrogen-based S-TENG (OHS-TENG) based on the Hofmeister effect and electrostatic interactions (Figure 3b) [69]. Using a one-pot method under UV light, a polyacrylamide (PAM)/PVA/NaCl hydrogel (AVN) is synthesized. To address the loss of conductivity caused by dehydration, the prepared hydrogel is immersed in a glycerol solution, where water molecules are replaced by glycerol molecules. This replacement mitigates water evaporation and retains ionic conductivity over time. The optimization process involved leveraging the Hofmeister effect and electrostatic interactions. The Hofmeister effect, which describes how different salts influence the hydration layer structure and ion migration within hydrogels, is used to select appropriate cation–anion combinations to significantly enhance ionic conductivity. Additionally, electrostatic interactions between ions and polymer chains within the hydrogel improved charge storage and transfer efficiency.

For practical applications in TENGs and flexible/wearable sensors, hybrid hydrogels with exceptional toughness, elasticity, conductivity, and multifunctionality serve as ideal materials. Long et al. developed a composite hydrogel (MPP–hydrogel) by combining conductive polymers with polymer networks (Figure 3c) [70]. The matrix structure utilized a double-network (DN) system formed via chemical crosslinking, enhancing mechanical properties. The first network is a chemically crosslinked PVA structure, while the second is composed of conductive polymers (e.g., polypyrene) for improved elasticity and conductivity. Two-dimensional Ti_3_C_2_Tx MXene (a two-dimensional (2D) transition metal carbide/nitride nanomaterial) nanosheets acted as hydrogen-bonding crosslinkers, significantly enhancing the flexibility and mechanical strength of the hydrogel. The resulting MPP–hydrogel membranes demonstrated resilience under various deformations, such as stretching and knotting, and quickly returned to their original shape without breaking. Its ability to self-repair from mechanical damage, such as scratches, further ensures its durability. This study highlights a promising flexible electronic material for applications in electronic skin and human–machine interaction.

Stretchability is a critical property for hydrogels used in self-healing TENGs. Zhang et al. developed a chemically crosslinked PAM/tannic acid (TA)/sodium alginate (SA)/MXene DN hydrogel with high viscoelasticity, ultrastretchability, and self-healing capabilities (Figure 3d) [28]. MXene, with its hydrophilic functional groups, promotes uniform dispersion and the formation of conductive networks within the hydrogel. Additionally, TA formed robust hydrogen bonds with PAM and SA, further improving mechanical properties. An Ecoflex encapsulation is employed to prevent electrode dehydration in the PAM/SA/TA/MXene hydrogel-based TENG (PTSM-TENG), achieving an output power density of 54.24 mW/m^2^. A smart glove incorporating PTSM-TENG employed PCA-PSO-SVM algorithms to classify and identify objects with an impressive accuracy of 98.7%. These results highlight the material’s potential in enhancing wearable device interactions and providing sustainable energy solutions.

Hydrogels with excellent mechanical properties are ideal for self-healing TENG applications. Luo et al. proposed a conductive hydrogel prepared through a fully physical crosslinked dual-network structure (Figure 3e) [71]. Borax crosslinked PVA chains via cis-diol complexes to form the first PVA-B network, while a second network is established via radical copolymerization of acrylamide (AM) and acrylic acid (AA). The resultant PVA/P(AM-co-AA) DN gel is immersed in FeCl_3_, where a complex metal-chelating structure further physically crosslinked the networks to yield PVA/P(AM-co-AA)-Fe^3+^ DN hydrogels. TENGs based on PVA/P(AM-co-AA)-Fe^3+^ electrodes exhibited excellent energy harvesting capabilities. The hydrogel exhibited high sensitivity to mechanical deformation, consistently outputting reliable electrical signals under various strain conditions. Its strain sensitivity displayed a good linear relationship, making it suitable for high-precision sensing applications. Furthermore, the dual-network structure endowed the hydrogel with high fatigue resistance, maintaining stable strain-sensing performance under prolonged cyclic stretching, proving its durability in dynamic and long-term monitoring scenarios.

### 2.3. Nanocomposites

In TENGs, the introduction of metallic nanoparticles (e.g., silver and gold) and carbon-based nanomaterials (e.g., graphene and carbon nanotubes) significantly enhances performance. Metallic nanoparticles, with their exceptional electrical conductivity and localized surface plasmon resonance effects, reduce contact resistance, improve charge transfer efficiency, and also contribute to rebuilding conductive networks in self-healing systems, enhancing both conductivity and self-repair properties. Meanwhile, graphene and carbon nanotubes, owing to their superior electrical conductivity, mechanical strength, and structural adaptability, have become integral components of TENGs. Graphene’s two-dimensional structure and exceptional conductivity are key to improving triboelectric output, while carbon nanotubes, with their high aspect ratio, facilitate the formation of continuous conductive networks, enhancing flexibility and stability. The integration of these nanomaterials into TENG systems addresses challenges in performance and durability. The synergistic integration of graphene and carbon nanotubes, or their combination with metallic nanoparticles, optimizes energy harvesting efficiency and expands applications in flexible electronics, wearable devices, and self-powered systems.

**Figure 4:**
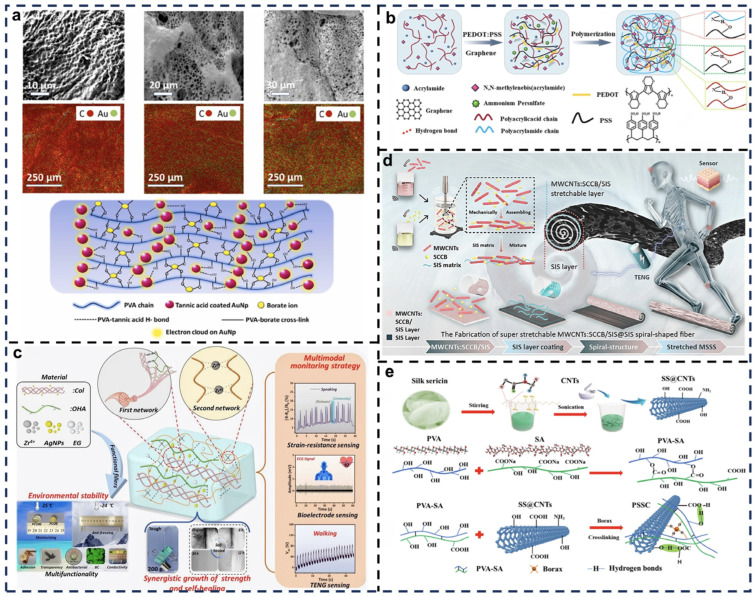
Nanocomposite materials applied to self-healing TENGs. (**a**) Preparation and performance analysis of PVA hydrogels. Reprinted with permission from Ref. [72]. Copyright 2022, Elsevier. (**b**) Synthesis process and optimization of MAGP hydrogels. Reprinted with permission from Ref. [73]. Copyright 2022, American Chemical Society. (**c**) Design and application of PCOBE collagen-based organic hydrogels. Reprinted with permission from Ref. [48]. Copyright 2023, Elsevier. (**d**) Experimental advancements in MSSS fibers. Reprinted with permission from Ref. [74]. Copyright 2022, Elsevier. (**e**) Fabrication methods and performance evaluation of PSSC hydrogels. Reprinted with permission from Ref. [75]. Copyright 2022, Elsevier.

Bagchi et al. developed a fully flexible, stretchable, self-healing, and semi-transparent TENG electrode by embedding gold nanoparticles (AuNPs) into a PVA hydrogel matrix. By incorporating AuNPs, the hydrogel achieved improved conductivity while retaining its stretchability, self-healing ability, and transparency (Figure 4a) [72]. These properties enabled the nanogenerator to efficiently convert mechanical energy into electrical energy under external forces while maintaining functionality after damage and subsequent self-repair. Furthermore, the semi-transparent nature of the film makes it suitable for wearable devices and flexible electronics. Testing showed that the device maintained high electrical output after multiple stretching and self-healing cycles, highlighting its reliability for long-term use in flexible electronic systems.

Materials with high conductivity and excellent physicochemical properties are ideal for self-healing TENG research. Dong et al. proposed a composite hydrogel material with integrated stretchability, adhesiveness, self-healing ability, and conductivity, which also exhibited adhesion to diverse materials such as glass, iron, wood, and ceramics (Figure 4b) [73]. This material is synthesized via free radical polymerization using acrylamide monomer (AAM), ammonium persulfate (APS) as an initiator, and N,N′-methylenebisacrylamide (MBA) as a crosslinker. The PAAM matrix contributed to flexibility and mechanical robustness. The incorporation of PVA as a second network introduced hydrogen bonds for dynamic crosslinking, enhancing toughness and stretchability through physical crosslinking via freeze–thaw cycles. Conductive components, such as ionic liquids (e.g., LiCl solutions), are introduced to improve ionic conductivity, forming ion conduction pathways within the hydrogel. This also lowered the freezing point, enabling stable conductivity and usability at low temperatures. The hydrogel exhibited stable conductivity under deformation, stretching, and damage.

The incorporation of metallic nanoparticles, such as silver and gold, into self-healing matrices improves the conductivity and energy-harvesting capabilities of TENGs. Song et al. developed a multifunctional dual-network organic hydrogel based on collagen, synthesized via a synergistic coordination strategy (Figure 4c) [48]. This hydrogel featured a primary network of self-assembled collagen and Schiff base bonds, complemented by a secondary network of reversible metal-coordination bonds between polyacrylic acid and Zr^4+^ ions dynamic crosslinking, including Schiff base bonds, collagen self-assembly, metal coordination, and hydrogen bonding, enabled the biomimetic preparation of the PCOBE hydrogel. This innovative strategy resulted in exceptional mechanical properties, conductivity, self-healing ability, and adaptability across a wide temperature range. PCOBE-TENG, after simple encapsulation, functioned as a self-powered sensor for monitoring human activities, facilitating comprehensive physiological monitoring and providing accurate health assessments for medical decision-making.

Carbon-based materials, with their exceptional conductivity, are commonly employed as TENG electrode components. Chung et al. designed a bioinspired, super-stretchable, dual-carbon conductive polymer fiber material specifically for health monitoring, energy harvesting, and self-powered sensors (Figure 4d) [74]. This design combined a polymeric matrix for mechanical flexibility with graphene and carbon nanotubes (CNTs) as conductive fillers. Graphene provided a high surface area and excellent conductivity, while CNTs enhanced mechanical strength and electron transport. TENGs fabricated using these fibers efficiently converted mechanical energy into electrical energy, leveraging the conductive and flexible properties of the material. Furthermore, the high conductivity and thermal stability of the dual-carbon materials allowed integration into thermoelectric systems for harvesting temperature gradient energy. These features make it highly adaptable for hybrid energy systems combining mechanical and thermal energy harvesting.

Despite advancements in TENG electrode materials, developing self-healing materials with tunable force-electric properties remains a challenge. Li et al. developed a hydrogel with tunable mechanical properties and self-healing capability based on SA and modified carbon nanotubes (MCNTs) for TENG applications in health monitoring, human motion sensing, and energy harvesting (Figure 4e) [75]. SA served as the primary hydrogel matrix, providing hydroxyl and carboxyl groups for active crosslinking sites. Modified CNT (e.g., carboxylation or aminated) improved compatibility with the hydrogel matrix, enhancing conductivity and mechanical performance. This combination resulted in a hydrogel with excellent mechanical tunability, self-healing properties, and conductivity, offering significant potential for flexible electronics and health monitoring applications. Its potential applications span wearable devices, flexible electronic skin, biomedical devices, and extreme environments, demonstrating significant practical value and versatility.

## 3. Structural Design in Self-Healing TENGs

### 3.1. Layered and Encapsulation Structures

Layered structures and encapsulation strategies are critical design elements for optimizing the performance of self-healing TENGs. Multilayered structures, which alternate triboelectric and conductive layers, maximize contact area and improve energy harvesting efficiency. These materials enable the rapid repair and functional recovery of damaged regions. This design enhances both mechanical stability and durability, crucial for long-term applications. Encapsulation strategies further boost the environmental adaptability of TENGs by adding protective layers, often self-healing elastomers, which shield active layers from moisture, oxidation, and external damage. These protective layers autonomously repair cracks or tears, ensuring the integrity and performance of triboelectric and conductive layers under repeated deformation. The combination of layered designs and encapsulation techniques ensures TENGs exhibit exceptional durability and reliability, particularly in applications like wearable devices, flexible electronics, and long-term energy harvesting.

**Figure 5:**
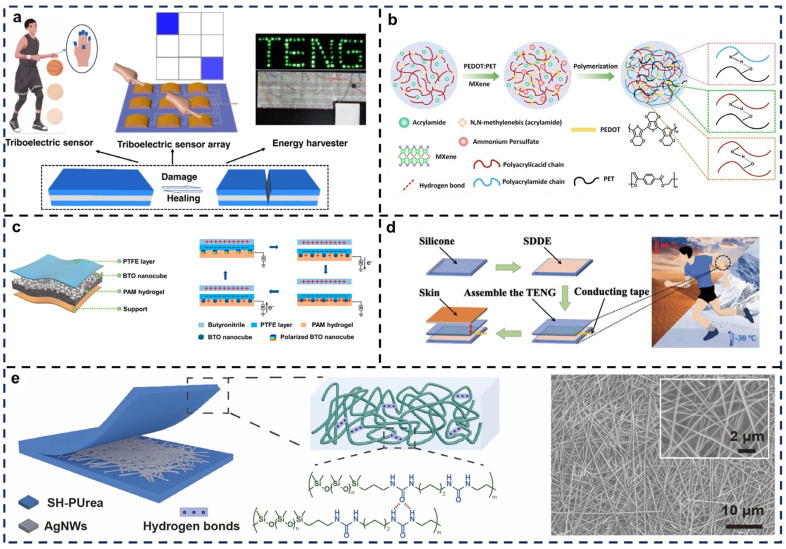
Layered and encapsulated structures of self-healing TENGs for artificial skin applications. (**a**) An energy harvester fabricated using an NBR/MXene/NBR film. Reprinted with permission from Ref. [50]. Copyright 2022, Elsevier. (**b**) Schematic representation of the preparation process of PPMP conductive hydrogel. Reprinted with permission from Ref. [76]. Copyright 2024, AIP. (**c**) Schematic of a sandwich-structured single-electrode TENG. Reprinted with permission from Ref. [77]. Copyright 2022, American Chemical Society. (**d**) Fabrication and demonstration of a TENG device based on SDDE. Reprinted with permission from Ref. [78]. Copyright 2022, Wiley. (**e**) Schematic of a sandwich-structured self-powered sensor composed of hydrogen-bond-based SH-PUrea and AgNW. Reprinted with permission from Ref. [79]. Copyright 2024, Elsevier.

He et al. proposed a multifunctional TENGs based on a flexible and self-healing sandwich-structured thin film (Figure 5a) [50]. The self-healing film structure comprises two layers of nitrile rubber (NBR) and a central layer of MXene. The sandwich structure’s flexibility allows it to conform to complex surfaces and accommodate dynamic deformation. The central MXene layer integrates electrode and triboelectric functions with self-healing capabilities, enabling the device to restore functionality after mechanical damage. Additionally, the high triboelectric performance of the surface layers and optimized design ensure the generation and efficient collection of high-density triboelectric charges. The energy harvester, fabricated using NBR/MXene/NBR films combined with polytetrafluoroethylene (PTFE) films, produces a peak-to-peak output voltage of 170 V and a current of 160 μA under cyclic compression from human hand motion. This multilayer design, combining flexibility, self-repairability, and high-performance triboelectric properties, provides a robust framework for multifunctional TENGs suitable for complex environments and long-term use. This innovative architecture highlights the potential of layered designs in advancing high-performance self-powered devices and flexible electronic applications.

Qin et al. proposed a dual-network hybrid PAAM/poly AA (PAA)/MXene/PEDOT (PPMP) hydrogel (Figure 5b) [76], which demonstrates the self-healing mechanism of the PPMP conductive hydrogel. A multifunctional TENGs based on this hydrogel is developed, comprising multiple functional layers. The layers include a conductive hydrogel electrode layer characterized by high flexibility and self-healing properties, a triboelectric layer to enhance output performance, and an encapsulation layer for environmental protection. The figure illustrates the detailed arrangement of these layers and their interactions, where the flexible encapsulation material protects the internal structure, ensuring moisture resistance, durability, and long-term reliability. This integrated design supports energy harvesting and enables real-time sensing applications, such as detecting the intensity and direction of dance movements.

Enhancing the output performance of TENGs remains a challenge. Wang et al. proposed a stretchable asymmetric piezoelectric BaTiO_3_ composite hydrogel for TENGs and multimodal sensors (Figure 5c) [77]. The asymmetric distribution of BaTiO_3_ particles, achieved through layered or gradient internal designs, optimizes charge separation and enhances triboelectric performance. The asymmetric piezoelectric composite hydrogel serves as the triboelectric layer, leveraging the piezoelectric and ionic conductivity properties to enhance output. This innovative structure integrates highly efficient piezoelectric particle distribution, a flexible hydrogel matrix, and dynamic crosslinking networks, offering a novel design for TENGs and multimodal sensors. Its multifunctionality and high stretchability represent significant advancements for flexible electronics and energy harvesting applications.

Achieving self-healing electronic skins capable of recovery under extreme conditions (e.g., high/low temperatures and humidity) remains challenging. Chou et al. developed a high-performance self-healing elastomer with a zwitterionic branched structure and bis-end aromatic disulfide design for use as a triboelectric material (Figure 5d) [78]. The material is applied in a TENG featuring exceptional environmental stability and self-healing properties. The TENG consists of a triboelectric layer made of the elastomer, offering excellent mechanical strength and self-healing capabilities; a conductive electrode layer for efficient charge collection; and a zwitterionic encapsulation layer providing waterproofing, corrosion resistance, and environmental stability. This integrated design ensures stable performance and reliability in extreme conditions, making it ideal for energy harvesting and self-powered sensing applications in harsh environments.

TENGs designed for extreme environments impose stringent requirements on electrode materials and overall structure. Dai et al. developed a self-healing triboelectric self-powered sensor featuring a triboelectric layer based on physically entangled self-healing polymers and silver nanowires (AgNW) encapsulated within a self-healing polymer as electrodes (Figure 5e) [79]. The sensor includes a self-healing substrate layer, a functional sensing layer, and an electrode layer, tightly integrated to optimize performance. The self-healing substrate layer utilizes a dynamic crosslinked polymer network capable of rapid self-repair via hydrogen bonding, ionic bonding, or dynamic covalent bonds, providing structural flexibility and support. This multilayer design enhances flexibility, allowing the sensor to conform to complex surfaces and sustain large deformations. Through the synergistic design of the dynamic crosslinked substrate, functional sensing, and conductive electrode layers, this flexible electronic skin demonstrates self-powered functionality, multifunctional sensing, and self-healing capabilities under various extreme conditions, presenting an innovative structural solution for flexible electronics and self-powered sensors.

### 3.2. Surface Microstructures and Porous Designs

The structural optimization of TENGs through surface engineering has significantly enhanced their energy-harvesting efficiency and durability. Introducing micro-patterns, such as pyramidal, conical, or ridged structures, onto the triboelectric layers significantly increases surface roughness and effective contact area, thereby improving charge separation efficiency during triboelectric interactions. Additionally, integrating microstructure surfaces with self-healing materials enables recovery from wear and ensures long-term performance stability. Furthermore, porous or wrinkled designs improve TENG flexibility and strain tolerance. Porous structures help distribute mechanical stress evenly, reducing the risk of localized damage, while wrinkled designs maintain stable energy output under deformation or stretching, making them ideal for wearable devices. When combined with self-healing capabilities, these designs allow automatic repair of micro-damages, ensuring consistent performance and high energy collection efficiency.

**Figure 6:**
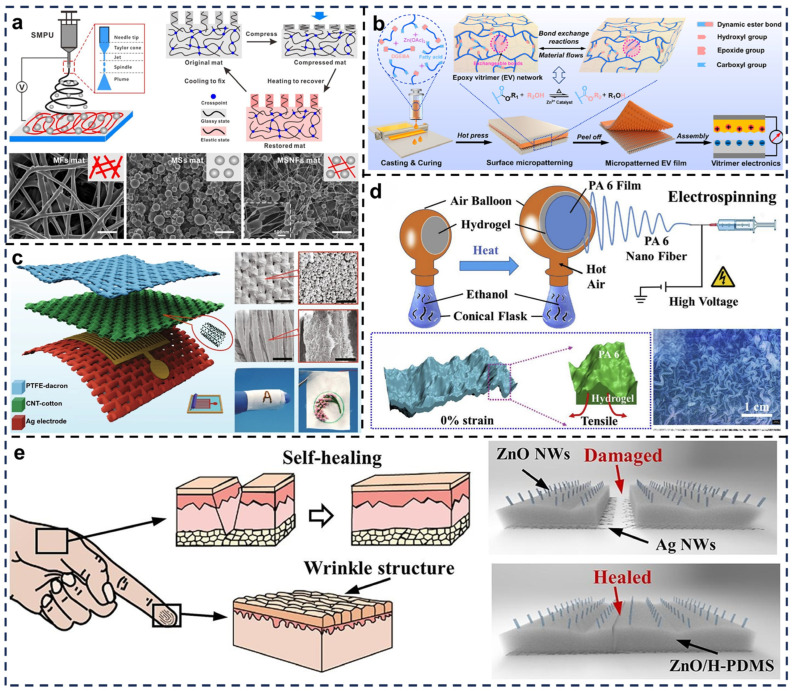
Applications of self-healing TENGs based on surface microstructures and porous design in electronic skin. (**a**) Tunable SMPU microarchitectures on the TENG surface achieved through electrospinning. Reprinted with permission from Ref. [80]. Copyright 2019, Elsevier. (**b**) Micro-patterned design of EV film as a triboelectric material. Reprinted with permission from Ref. [81]. Copyright 2023, Elsevier. (**c**) Structural design of F-TENG incorporating CNT molecules. Reprinted with permission from Ref. [82]. Copyright 2021, Springer Nature. (**d**) Hierarchically wrinkled triboelectric PA6 films. Reprinted with permission from Ref. [83]. Copyright 2019, Elsevier. (**e**) Development of ZnO NWs/H-PDMS hierarchical wrinkled structures inspired by human skin. Reprinted with permission from Ref. [84]. Copyright 2024, Elsevier.

Xiong et al. addressed the environmental adaptability challenges of traditional TENGs in underwater applications by developing a multifunctional TENG using electrospinning-based shape-memory polyurethane (SMPU) (Figure 6a) [80]. The TENG structure comprises three scalable microarchitectures: microfibers (MF), microspheres (MS), and microsphere–nanofiber (MSNF) mats, which demonstrate exceptional self-recovery capabilities at macro and micro scales. The introduction of tunable microstructure designs significantly enhances the surface contact area through optimized geometric configurations, effectively boosting triboelectric efficiency. Moreover, the adjustable microstructure parameters allow precise control over output performance. As a result, this multifunctional TENG achieves stable and efficient energy output while demonstrating improved durability and adaptability for energy harvesting and temperature sensing in complex aquatic environments.

Zhang et al. innovatively designed a self-healing and recyclable TENG using a microporous surface structure based on epoxy trimer (EV) (Figure 6b) [81]. The material’s unique properties facilitate the formation of microporous structures through dynamic covalent bonding, enabling bond exchange reactions under specific conditions for specialized processing and performance tuning. Fabricated via hot-pressing techniques, the material exhibits smooth pore walls, uniformly distributed pores ranging from nanometer to micrometer scales, and increased specific surface area. These features enhance mechanical properties, improve triboelectric effects, and provide efficient charge transport pathways. These features not only enhance mechanical properties but also improve triboelectric effects and provide efficient charge transport pathways. Additionally, the incorporation of dynamic covalent bonds ensures stability and self-healing capabilities, making this design ideal for high-performance electronic applications.

To address the limitations of conventional interactive devices that rely on external power sources, Yi et al. proposed a fully fabric-based TENG (F-TENG) tailored for smart wearable devices and flexible electronics (Figure 6c) [82]. This TENG comprises silver-coated fabric combined with CNTs and PTFE-coated textiles, demonstrating exceptional energy harvesting performance. The F-TENG achieved a peak instantaneous power density of 170 μA/m^2^ and successfully illuminated 38 LEDs with an area of 2 × 2 cm^2^, showcasing its practical application potential. This innovative fabric-based design highlights the feasibility of integrating TENGs into wearable devices for sustainable energy solutions.

Qi et al. reported a wearable, high-performance TENG with an easily manufacturable design, partially demonstrated in (Figure 6d) [83]. Using a conical flask, ethanol evaporation under heating prepared the conditions for subsequent nanofiber fabrication. Polyamide 6 (PA6) nanofibers are formed via electrospinning, where high voltage and hot air facilitate the creation of a unique nanofiber membrane surface. Although explicit microporous structures are not specified, the layered wrinkles likely imply microporous characteristics. This surface structure enhances the frictional area, improving charge generation efficiency. Under tensile strain, the material adapts to deformation while maintaining electrical performance, ensuring stability and efficiency in wearable applications.

To address the rapid reduction of micro/nanostructured interfaces in self-healing materials, Lui et al. proposed a heterogeneous interface coupling strategy to fabricate a stable layered wrinkle structure for self-healing TENGs (S-TENG) (Figure 6e) [84]. Inspired by human skin, the S-TENG incorporates materials with self-healing and antibacterial properties, mimicking the skin’s structure and functionality. This TENG autonomously repairs damage while resisting bacterial infections, providing high sensitivity, durability, and improved environmental adaptability.

## 4. Applications in Artificial Skin

### 4.1. Healthcare and Tactile Sensing

With the rapid development of TENG technology, its potential applications in wearable devices, electronic skins, Internet of Things (IoT) sensors, and smart healthcare are becoming increasingly evident. TENGs exhibit immense promise in health monitoring and human–machine interaction despite facing practical limitations. Researchers are optimizing materials and structures to enhance TENG sensitivity, flexibility, multifunctionality, and adaptability for various scenarios. These advancements aim to address challenges in smart healthcare, remote monitoring, and emergency care by improving device efficiency and reliability. As material science and structural design continue to evolve, TENGs are poised to become core technologies driving advancements in smart electronics, health monitoring, and IoT applications.

**Figure 7:**
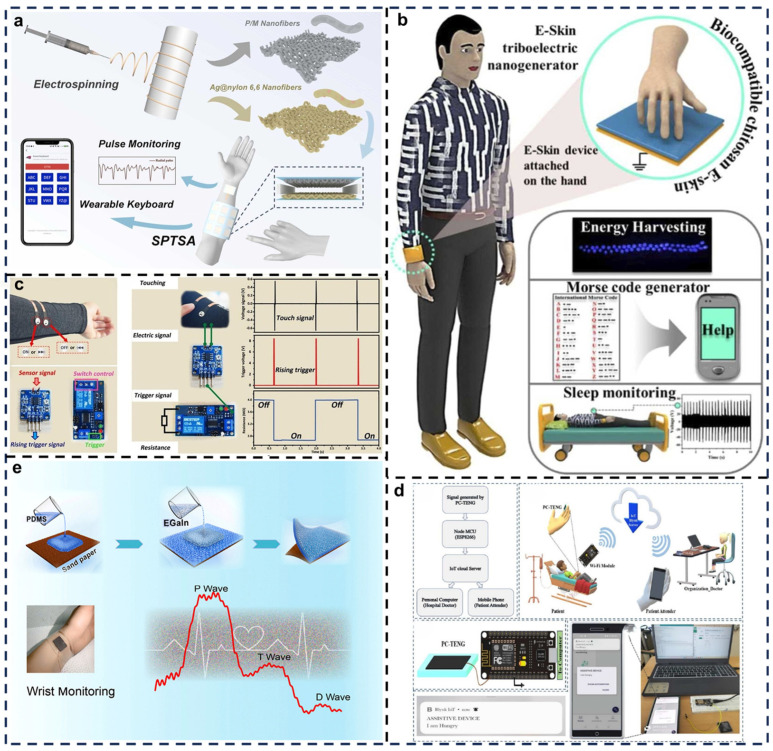
Applications of self-healing TENGs in healthcare and tactile sensing devices. (**a**) Schematic illustration of the fabrication and application of a self-powered tactile sensing array (SPTSA). Reprinted with permission from Ref. [85]. Copyright 2023, Elsevier. (**b**) Applications of TENG devices based on CS-glycerol composites. Reprinted with permission from Ref. [54]. Copyright 2024, Elsevier. (**c**) Tactile sensors based on PFL@WFCF-TENG for human–machine interface (HMI) scenarios. Reprinted with permission from Ref. [55]. Copyright 2021, Elsevier. (**d**) IoT-assisted devices based on PC-TENG technology. Reprinted with permission from Ref. [86]. Copyright 2024, Elsevier. (**e**) Ultra-sensitive triboelectric tactile sensors applied in human pulse monitoring. Reprinted with permission from Ref. [56]. Copyright 2021, Elsevier.

Electronic skins are often used for mechanical energy harvesting and tactile sensing. Zhi et al. designed a biocompatible, antibacterial, all-fabric TENG using electrospun nanofibers (Figure 7a) [85]. This self-powered tactile sensor consists of two triboelectric layers and two electrodes. The tribometries layer is made of P/M nanofiber membranes doped with chitin–glycerol nanosheets, which enhance the β-phase content of polyvinylidene fluoride (PVDF) nanofibers, thereby optimizing the TENGs output performance. The triboelectric-positive layer comprises Ag nanoparticles modified nylon 6,6 (PA66-Ag NPs) fiber membranes embedded with antibacterial Ag nanoparticles. These layers are supported by conductive fabric membranes, which serve as the upper and lower electrodes, and foam tape spacers that form microstructures to enhance tactile sensitivity. The resulting TENG is suitable for mechanical energy harvesting and tactile sensing, with potential applications in pulse monitoring and wearable keyboards. Additionally, a tactile sensor array built with this TENG has demonstrated promising applications in smart wearable keyboards, intelligent locks, and high-resolution tactile mapping, highlighting its versatility in human–machine interaction.

Electronic skins mimic the functions of natural skin. Rani et al. developed a multifunctional e-skin device based on TENG, utilizing biocompatible chitosan films as active layers and gold as electrodes (Figure 7b) [54]. This e-skin can serve as a Morse code transmitter for smart healthcare applications. The biocompatible materials ensure safety, while the e-skin generates triboelectric signals through contact with the skin surface, enabling Morse code detection and transmission. This design facilitates remote information communication and real-time health monitoring, offering practical solutions for emergency communication and smart healthcare. The design highlights the potential of TENG technology in smart healthcare, health monitoring, and emergency communication, offering new insights for future edible and skin-attachable electronic devices.

Textile-based TENGs have gained attention for wearable power sources and motion monitoring, though their output performance and environmental adaptability in single-electrode modes remain suboptimal. Wang et al. developed a moisture-resistant, stretchable single-electrode fabric TENG composed of a porous flexible layer and a waterproof conductive fabric (Figure 7c) [55]. Demonstrations of this TENG showed stable performance under humidity variations and during stretching or deformation, confirming its flexibility, reliability, and potential for tactile sensing applications. The device effectively supports self-powered pulse monitoring and gesture detection on wearable devices. Beyond mechanical energy harvesting, this TENG offers multifunctional tactile sensing capabilities, making it suitable for smart wearable devices in humid environments, with applications spanning portable computing peripherals, smart cities, robotics, and security systems.

In an era of rapid technological development, self-powered sensors play a key role in wearable technology and IoT systems. Viswanathan et al. designed a skin-conformal TENG operating in single-electrode mode, driven by template-printed carbon electrodes (Figure 7d) [86]. Energy harvested by the TENG not only powers sensing components but also facilitates wireless communication within IoT networks. This system demonstrated its utility as a self-powered Morse code generator and a remote tactile sensing patch for elderly care. This system captures human motion energy via triboelectric effects, using a self-powered Morse code generator for signal transmission. Tactile information is remotely transmitted to caregivers or medical teams, enabling real-time monitoring and health management. Particularly suited for elderly care, this system supports safe home-based monitoring, emergency alerts, and health tracking. Future integration with machine learning algorithms could advance patient activity analysis and predictive health monitoring.

Self-powered tactile sensors hold great potential for health monitoring and motion performance detection, yet manufacturing stretchable, flexible, and highly sensitive sensors remains challenging. Wang et al. developed a simple, high-performance flexible triboelectric pressure sensor designed for ultra-low-pressure detection (Figure 7e) [56]. The sensor features gallium–indium alloy (EGaIn) electrodes, which provide excellent conductivity and stretchability, ensuring stable electrical signal generation under applied pressure. Triboelectric layers produce charge separation under mechanical stimuli, generating electrical signals for detecting minute external pressures. This design is particularly suited for precise tactile sensing in ultra-low-pressure environments, with applications in flexible electronics, light-touch monitoring, and human–machine interactions.

### 4.2. Human–Machine Interaction

Human–machine interaction (HMI) has become a focal point of research in recent years, propelled by the rapid advancements in TENG technology. However, structural and material limitations hinder the broader application of TENGs in HMI. To address these challenges, researchers have focused on improving TENG materials, particularly through innovative applications of specific materials, which have significantly driven advancements in HMI technologies and their auxiliary functionalities. The development of self-healing TENGs has been particularly impactful, enabling self-repair, self-powering, flexible sensing, and intelligent feedback. These capabilities support efficient energy harvesting and precise interaction sensing across diverse application scenarios. This innovation has spurred the development of smart wearable devices and HMI technologies. Future progress in TENG will continue to enhance energy harvesting efficiency, sensing accuracy, multifunctionality, and environmental adaptability, driving HMI technology toward greater intelligence, sustainability, and personalization.

**Figure 8:**
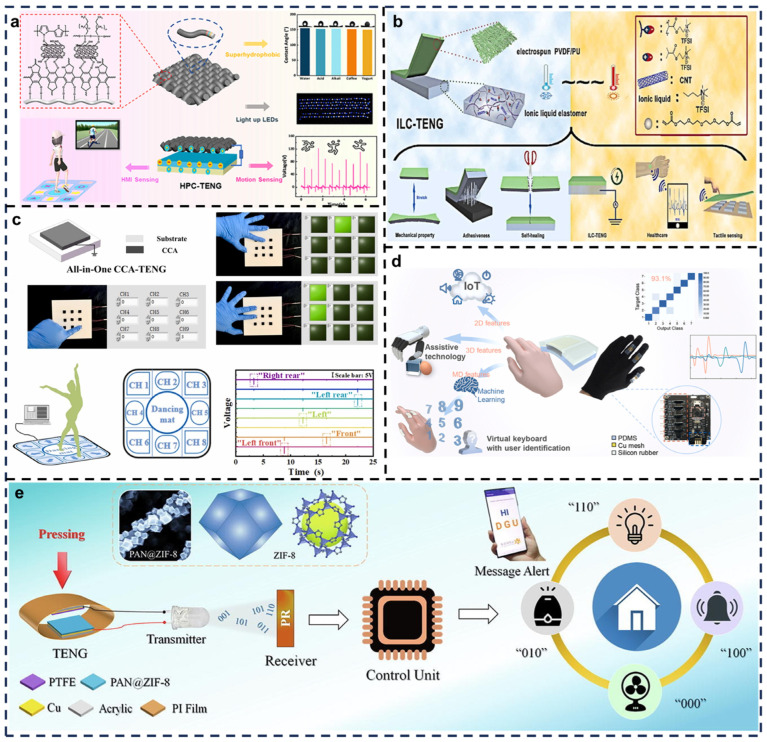
Self-healing TENG applied in human–machine interaction scenarios. (**a**) Multi-channel sensing of HPC-TENG in various scenarios. Reprinted with permission from Ref. [57]. Copyright 2023, American Chemical Society. (**b**) Structural and multifunctional schematic of ILC-TENG. Reprinted with permission from Ref. [87]. Copyright 2022, Elsevier. (**c**) Application of human–machine interface sensors based on CCA-TENG. Reprinted with permission from Ref. [58]. Copyright 2022, Elsevier. (**d**) BA-TENG used in various HMI scenarios. Reprinted with permission from Ref. [88]. Copyright 2021, Elsevier. (**e**) Self-powered VLC system driven by PZ-TENG. Reprinted with permission from Ref. [59]. Copyright 2022, Elsevier.

The rapid development of TENGs has garnered significant attention for self-powered, flexible, and wearable electronics. He et al. introduced an innovative TENG constructed from specially treated cotton fabric (Figure 8a) [57]. The fabric achieved conductivity and superhydrophobicity through multiple modifications, including polydopamine (PDA), CNTs, PPy, and hydrophobic treatment with cetyltrimethoxysilane (HDTMS). This intricate structure allows the fabric to function simultaneously as a triboelectric layer and an electrode, simplifying the design and improving the durability of the TENG. Beyond energy harvesting, this TENG has been applied in interactive systems such as smart gaming mats that track user movements. Such applications highlight its potential in fields like sports, gaming, entertainment, and healthcare monitoring. Tests demonstrated the fabric’s resistance to water, wear, and penetration, along with hydrophobicity under chemical exposure (e.g., acids and alkalis). Additional tests confirmed its self-cleaning properties for dust removal and breathability, demonstrated via hydrochloric acid permeability experiments, affirming its suitability for wearable applications.

With the rapid progress of IoT and AI, electronic skin devices have gained significant attention, though the stretchability of triboelectric layers remains constrained. Cheng et al. developed a stretchable, flexible, self-healing, environmentally stable, and durable ionic liquid elastomer-based triboelectric generator (ILC-TENG) for energy harvesting, motion sensing, healthcare monitoring, and tactile recognition (Figure 8b) [87]. This triboelectric electronic skin exhibits exceptional mechanical strength, stretchability, self-adhesiveness, and environmental adaptability, enabling self-powered health monitoring and tactile sensing. Its high durability and flexibility allow it to conform to body contours and function reliably under various environmental conditions. The device is capable of real-time monitoring of physiological signals, such as heartbeat and respiration, while also providing tactile feedback. These features make it highly suitable for applications in smart healthcare, wearable health monitoring devices, and HMI systems. Its self-adhesive and environmentally robust properties further enhance its adaptability and stability.

Significant advancements in HMI research have been achieved with the introduction of TENG technology. Wang et al. developed a novel cellulose carbon nanotube aerogel-based TENG (CCA-TENG) with high output, moisture resistance, structural simplicity, and biodegradability for environmental energy harvesting and self-powered sensing (Figure 8c) [58]. The CCA serves as both the triboelectric layer and electrode, featuring a 3D porous structure, high specific surface area, and enhanced dielectric properties. Leveraging these advantages, multi-unit CCA-TENGs enable multichannel pressure monitoring to track human or animal activity in specific environments. Additionally, the sensor integrates a counting function to record activation frequency over time. In dance mat scenarios, each direction corresponds to a channel in the HMI sensor, where dancer movements generate electrical signals displayed on an interactive interface to evaluate step intensity and position. This research showcases the immense potential for HMI systems, smart wearables, self-powered wireless sensing, and multichannel pressure sensing.

Despite substantial progress in HMI, certain limitations remain. Novel HMIs directly expressing human intentions have become essential solutions, leveraging alternative human characteristics. Among these, HMIs based on finger movements have gained attention for their high precision and multi-degree-of-freedom control. Luo et al. designed a simple, high-resolution bending angle triboelectric sensor to construct a glove-based multidimensional HMI (Figure 8d) [88]. The smart glove integrates TENG sensors that generate electrical signals upon finger bending, enabling real-time detection of finger motion. The sensor’s high sensitivity to triboelectric effects facilitates precise gesture recognition, supporting advanced applications such as robotic control, virtual reality, and augmented reality interactions. This innovative design demonstrates broad application potential in intelligent control and wearable devices.

Recent advancements in HMI technologies have led to increasing integration of novel approaches. Pandey et al. proposed a TENG utilizing zeolitic imidazolate framework nanocrystal-decorated electrospun polyacrylonitrile (PAN) nanofibers as high-performance triboelectric materials. They demonstrated a self-powered visible light communication (VLC) system for wireless HMI applications, addressing VLC power sustainability issues (Figure 8e) [59]. Triboelectric energy drives the VLC system through self-powering mechanisms, with optical signals converted to data for wireless transmission. This system captures human movements and transmits them via visible light signals, enabling remote control and intelligent interaction. Potential applications include remote personal identification, security defense, and smart home authentication and monitoring systems. This innovation underscores the potential of combining triboelectric energy harvesting with advanced communication technologies for future intelligent systems.

### 4.3. Robots and Motion Detection

The rapid development of TENGs has ushered in new opportunities for research in robotic sensing and intelligent motion detection. TENGs demonstrate great potential in smart robotic sensing, such as in wearable sensors to detect and measure various human physiological signals (e.g., heart rate, respiration, body temperature). However, challenges remain in ensuring stable output performance and improving energy conversion efficiency. TENGs also enable object presence and motion sensing, enhancing robots’ abilities to navigate and interact effectively with their environment. Bio-inspired designs have further improved adaptability for motion monitoring by incorporating self-healing and antifreeze materials, enhancing durability and expanding application potential. Integrated with advanced sensor technologies, TENG applications are expanding, fostering progress in intelligent robotics and motion monitoring systems while laying the groundwork for applications in smart manufacturing, sports training, and medical prosthetics.

**Figure 9:**
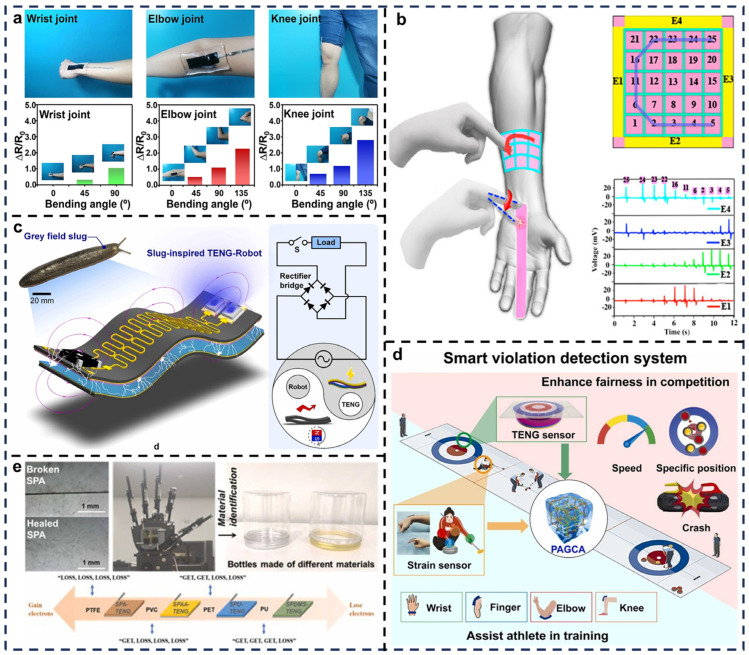
Applications of self-healing TENGs in robotics and motion detection. (**a**) MF-TENGs worn on multiple joints as motion detection devices. Reprinted with permission from Ref. [89]. Copyright 2021, American Chemical Society. (**b**) SFTS patches for robotic motion control. Reprinted with permission from Ref. [60]. Copyright 2018, American Chemical Society. (**c**) Snail-inspired bionic TENG-robot. Reprinted with permission from Ref. [90]. Copyright 2022, Elsevier. (**d**) Smart violation detection system based on TENG sensors. Reprinted with permission from Ref. [61]. Copyright 2024, Elsevier. (**e**) Robotic hand with triboelectric sensing arrays for material type recognition. Reprinted with permission from Ref. [62]. Copyright 2024, Elsevier.

Yang et al. developed a single-electrode multifunctional TENG (MF-TENG) using a PVA-based hydrogel with polydopamine-modified CNTs (PDA-CNTs) and borate compounds (Figure 9a) [89]. This composition endowed the material with high conductivity and reversible self-healing properties, allowing rapid repair at room temperature while maintaining the original electrical output. The MF-TENG generates triboelectric charges using human motion, with output signals produced through a single-electrode mode for motion monitoring. When attached to joints like wrists, knees, or elbows, it detects motion range and health status through resistance changes. Notably, the device achieves recovery in 10 min at room temperature without a significant impact on electrical performance. This device integrates health monitoring and photothermal therapy, showing promise in wearable applications for soft robotics, motion health monitoring, and rehabilitation.

Wearable sensors have been a focal point of research, especially for integrating robotics with motion tracking. Chen et al. introduced a self-powered, flexible triboelectric sensor (SFTS) patch for finger trajectory sensing (Figure 9b) [60], leveraging collected data for robotic control. Made from flexible and eco-friendly materials, this low-cost sensor offers potential in robotic control, touchscreens, and electronic skins. The SFTS array detects finger touch positions and forces, generating voltage signals transmitted to control systems for robotic arm motion. By analyzing touch positions and voltage changes, the signals are translated into 3D motion instructions, enabling precise HMI. This innovative approach offers solutions for remote control and precise positioning, with promising applications in wearable devices, robotic motion control, and virtual reality interactions.

Biological evolution has inspired specialized structures and skills for environmental adaptation. Biomimetic robots exploit soft and compliant designs for enhanced adaptability and self-protection in complex scenarios. Peng et al. developed a snail-inspired magnetic soft microrobot fully integrated with a TENG module (snail-inspired TENG-robot) for onboard sensing and self-powered charging (Figure 9c) [90]. Driven by an unrestrained rotating magnetic field, the robot produces a pedal wave sequence through periodic body undulation, enabling forward motion across diverse terrains (e.g., plains, slopes, gaps, and tunnels). This innovative design demonstrates superior environmental adaptability, remote controllability, and motion efficiency, holding significant potential for biomedical applications. Figure 9c illustrates the robot’s structure and circuit diagram, showcasing how the TENG module generates power through triboelectric effects. Bionic demonstrations, such as luminescence, self-charging, and temperature sensing, validate the robot’s multifunctionality and provide new design pathways for soft robotics.

Conventional foul detection systems rely on referees and high-frame-rate cameras, limiting accuracy in dynamic scenarios. Tian et al. developed an intelligent foul detection system with self-powered, self-healing, and antifreeze properties (Figure 9d) [61], using a nanocomposites hydrogel (PAGCA) l as the electrode material in single-electrode TENGs. With PDMS as the negative electrode, this system functions reliably in low temperatures (e.g., −30 °C), meeting the unique demands of ice sports. It detects the speed, position, and collisions of sports equipment (e.g., curling stones) and monitors joint activities during training. This system provides precise feedback to athletes, enhancing training outcomes, and offers the potential for real-time foul detection in other sports.

Frequent material contact during identification processes exposes sensor arrays to mechanical damage. Cheng et al. developed a self-healing triboelectric sensor array using self-healing polyamide (SPA) (Figure 9e) [62]. The array integrates triboelectric units with varying electron affinities (self-healing polyacrylic acid copolymer, self-healing polyurethane, and self-healing polysiloxane) to create a fingerprint-like waveform system capable of precise material identification. Application tests demonstrated accurate recognition of daily plastic items, with characteristic waveforms reflecting unique electron affinity sequences (e.g., “GET” and “LOSS” patterns). This technology showcases potential in material recognition for applications in smart robotics, prosthetics, and automated manufacturing systems.

## 5. Challenges and Future Directions

### 5.1. Challenges

One of the primary challenges in the development of self-healing TENGs for artificial skin lies in material limitations. Current self-healing materials often exhibit trade-offs between mechanical strength and healing efficiency, with highly flexible materials sometimes lacking durability or electrical performance. Additionally, maintaining self-healing properties in diverse environmental conditions, such as extreme temperatures or high humidity, remains difficult. A second challenge involves integrating multifunctionality without compromising performance. While TENGs aim to combine energy harvesting, tactile sensing, and other functionalities, the simultaneous optimization of these properties can lead to complex designs that are challenging to scale up for commercial use. Another significant obstacle is long-term stability and reliability. Repeated damage and repair cycles, along with exposure to external factors such as wear, corrosion, and fatigue, can degrade the performance of self-healing TENGs over time. Ensuring consistent functionality across multiple cycles of self-repair and environmental stresses remains a critical issue. Furthermore, biocompatibility, particularly for artificial skin applications, presents another challenge. Materials must ensure safety and compatibility with human tissue while maintaining their performance, which may involve addressing antibacterial properties to prevent infections during long-term use. Finally, fabrication and cost remain hurdles for widespread adoption. The synthesis of advanced self-healing materials and the implementation of complex structural designs often involve high costs and time-intensive processes, limiting their practicality for large-scale production. Moreover, understanding and optimizing the conditions required for self-healing, such as specific temperature or humidity levels, remains an area needing further exploration.

### 5.2. Future Directions

Future research should prioritize the development of advanced self-healing materials with tailored properties to balance flexibility, strength, and electrical performance. Innovations in material design could enhance both durability and healing speed under diverse environmental conditions. Biocompatibility should also be emphasized, especially for artificial skin applications, by incorporating antibacterial properties into the material design to maintain hygiene and safety. Exploring optimal healing conditions and providing quantitative benchmarks for healing speed will further support the practical implementation of self-healing TENGs. Efforts should also focus on creating scalable and simplified designs for multifunctional TENGs. Modular architectures that integrate energy harvesting, sensing, and other functionalities could facilitate commercialization. Research into environmental adaptability, such as maintaining self-healing and triboelectric properties under extreme conditions, will also help broaden the application scope of these devices. Finally, improving the affordability and sustainability of TENGs by using bio-based or recyclable materials and optimizing fabrication processes will be critical for their broader adoption in wearable electronics and robotics.

## Data Availability

No new data were created.
